# DNA methylation status of nuclear-encoded mitochondrial genes underlies the tissue-dependent mitochondrial functions

**DOI:** 10.1186/1471-2164-11-481

**Published:** 2010-08-19

**Authors:** Masaki Takasugi, Shintaro Yagi, Keiji Hirabayashi, Kunio Shiota

**Affiliations:** 1 Laboratory of Cellular Biochemistry, Department of Animal Resource Science/Veterinary Medical Sciences, the University of Tolyo, Tokyo 113-8657, Japan; 2 National Institute of Advanced Industrial Science and Technology, Tsukuba, Ibaraki 305-8561, Japan

## Abstract

**Background:**

Mitochondria are semi-autonomous, semi-self-replicating organelles harboring their own DNA (mitochondrial DNA, mtDNA), and their dysregulation is involved in the development of various diseases. While mtDNA does not generally undergo epigenetic modifications, almost all mitochondrial proteins are encoded by nuclear DNA. However, the epigenetic regulation of nuclear-encoded mitochondrial genes (nuclear mt genes) has not been comprehensively analyzed.

**Results:**

We analyzed the DNA methylation status of 899 nuclear mt genes in the liver, brain, and heart tissues of mouse, and identified 636 nuclear mt genes carrying tissue-dependent and differentially methylated regions (T-DMRs). These nuclar mt genes are involved in various mitochondrial functions and they also include genes related to human diseases. T-DMRs regulate the expression of nuclear mt genes. Nuclear mt genes with tissue-specific hypomethylated T-DMRs were characterized by enrichment of the target genes of specific transcription factors such as FOXA2 in the liver, and CEBPA and STAT1 in the brain.

**Conclusions:**

A substantial proportion of nuclear mt genes contained T-DMRs, and the DNA methylation status of numerous T-DMRs should underlie tissue-dependent mitochondrial functions.

## Background

Mitochondrial dysfunction is a common cause of human diseases [1,2], and thus understanding the regulation of mitochondrial functions is critical. Mitochondria do not contain histones [3], and almost all mtDNA is unmethylated [4,5], indicating that mtDNA is not epigenetically regulated. However, while mammalian mitochondria are estimated to consist of more than 1,500 proteins, only 13 proteins are encoded by mtDNA.

Methylation of nuclear DNA is a major component of epigenetic system in mammalian cells, and is involved in silencing of gene transcription and maintaining genomic stability [6,7]. Hypomethylation of regulatory regions is required to allow expression of genes [8,9]. Microarray-based DNA methylation analysis revealed the existence of thousands of tissue-dependent and differentially methylated regions (T-DMRs) in the mouse and human genomes [10,11]. While the T-DMRs of some genes, such as *Oct-4 *and *Nanog*, are hypomethylated only in a few cells [12,13], the methylation status of most T-DMRs is not specific, but common to certain cells or tissues [11,14]. Tissue-dependent methylation status of T-DMRs, including tissue-specific methylation status of T-DMRs, forms a distinctive DNA methylation profile for each cell type [8,11,15].

A nuclear mt gene, *Ant4*, which encodes mitochondrial outer membrane protein, contains T-DMRs which is specifically hypomethylated in the testis [16,17]. Also, few dozens of nuclear mt genes in mice are hypomethylated in the liver relative to the cerebrum [11]. However, the presence of T-DMRs in nuclear mt genes has not been comprehensively analyzed; this is necessary for understanding the regulation of mitochondrial functions. In this study, we analyzed the DNA methylation of 899 nuclear mt genes in the liver, brain, and heart tissues of mouse; these tissues consume large amounts of energy and are highly susceptible to mitochondrial dysfunctions. Our results indicated that at least 636 nuclear mt genes, which account for 71% of the total investigated nuclear mt genes, contain T-DMRs in their transcription start site (TSS) flanking regions (-7~+3 kb of TSSs), and that the differential methylation status of these T-DMRs is associated with tissue-dependent mitochondrial functions.

## Results and Discussion

### Identification of T-DMRs in the TSS flanking regions of nuclear mt genes in the liver, brain, and heart tissues

**Figure 1:**
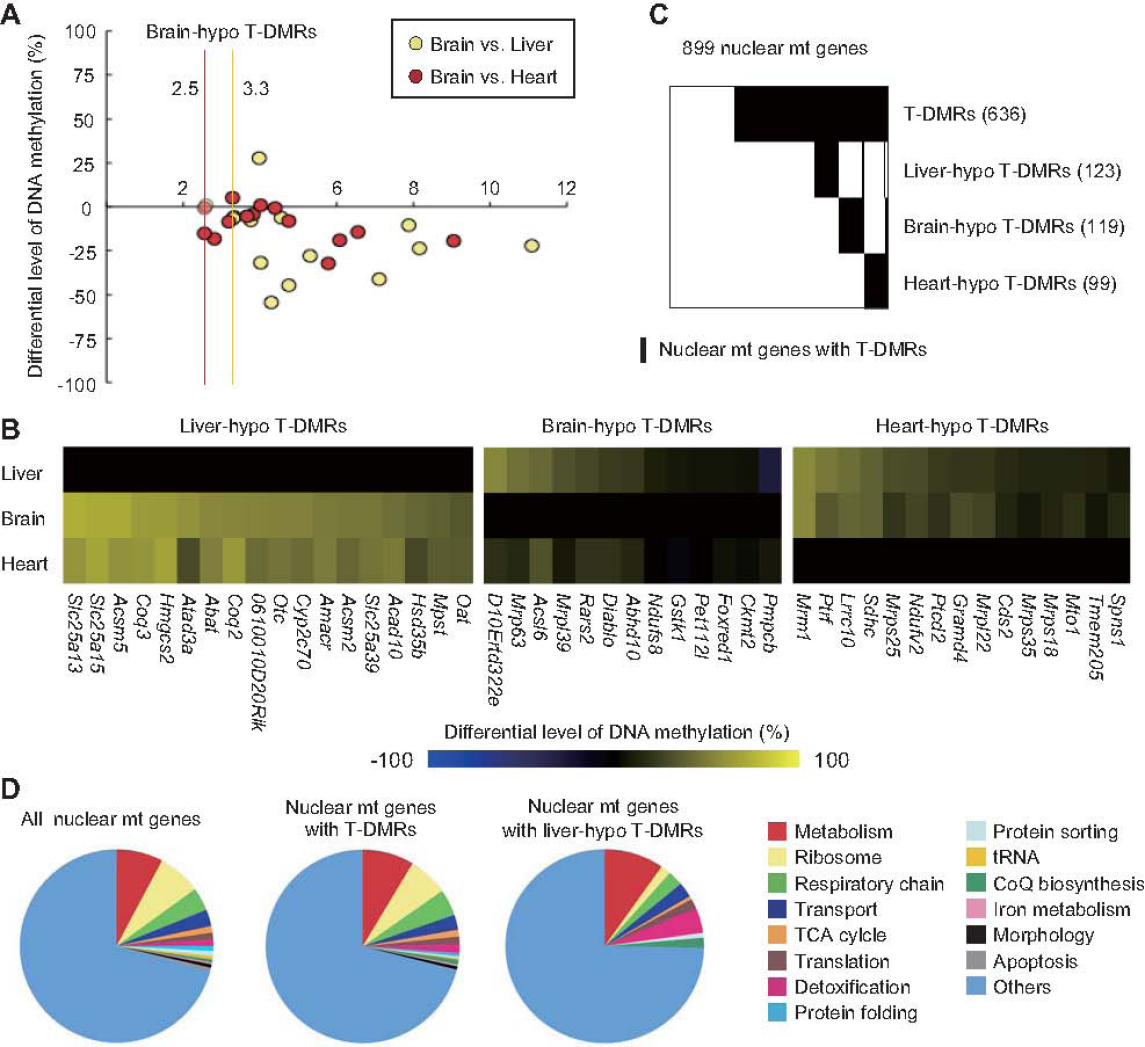
**Nuclear mt genes with T-DMRs identified by D-REAM analysis**. (A) Differential methylation levels of HpyCH4IV sites for which the relative hypomethylation levels were analyzed using the D-REAM analysis. For each HpyCH4IV site, the corresponding MATscore is indicated along the horizontal axis. In each tissue comparison, the lowest MATscore of the HpyCH4IV sites whose hypomethylation were confirmed using COBRA, was considered as a threshold value for detecting hypomethylated regions in the tissue comparison. Pale-colored circles indicate that the corresponding HpyCH4IV site was not included in the hypo T-DMRs (see text for definition). (B) Matrices show the differential level of DNA methylation of the liver-, brain-, and heart-hypo T-DMRs in the tissues relative to the respcective methylation level of hypo T-DMRs in the liver, brain, and heart, respectively. Each column represents a different hypo T-DMR. The names of nuclear mt genes containing hypo T-DMRs in their TSS flanking regions are displayed under each column. (C) Number of nuclear mt genes with T-DMRs. Matrix columns represent different nuclear mt genes. (D) Function of nuclear mt genes, nuclear mt genes with T-DMRs and nuclear mt genes with liver-hypo T-DMRs. Pie charts illustrate the percentage of genes that possess the indicated functions.

To investigate the DNA methylation status of nuclear mt genes in the liver, brain (cerebrum), and heart tissues, we conducted pairwise tissue comparisons using model-based analysis of tiling-array (MAT) along with D-REAM analysis [11,18]. Differences in the DNA methylation status at HpyCH4IV sites (ACGT sites) were exhibited as differences in MATscores of the probes corresponding to the selectively amplified fragments generated by digestion of unmethylated HpyCH4IV sites [11]. We identified tissue-dependent and differentially methylated HpyCH4IV sites within the 10 kb-TSS flanking regions of 899 RefSeq genes that are known to encode mitochondrial proteins and are registered in the Mitop2 database as a reference set [19]. In each tissue comparison, HpyCH4IV sites with MATscores above a specific threshold value were identified as hypomethylated T-DMRs in that particular tissue. The lowest MATscore of HpyCH4IV sites whose hypomethylation were confirmed by combined bisulfite restriction analysis (COBRA) (Figure 1A,B and Additional file 1,2), were considered as the threshold values of MATscore. COBRA was performed for HpyCH4IV sites whose MATscores were larger than 2 when one tissue was compared to the either of the other 2 tissues. We identified T-DMRs in the 10 kb-TSS flanking regions of 636 nuclear mt genes (Figure 1C and Additional file 3), with 123, 119, and 99 nuclear mt genes with T-DMRs specifically hypomethylated in the liver, brain, and heart tissues, respectively, when compared with the other 2 tissues (hereafter referred to as tissue-hypo T-DMRs) (Figure 1C and Additional file 4).

Most functional categories of mitochondria classified by Mitop2 were found in nuclear mt genes with T-DMRs and with hypo T-DMRs (Figure 1D). Overrepresentation and underrepresentation of nuclear mt genes with liver-hypo T-DMRs were found in the detoxification and mitochondrial ribosomal categories, respectively (*P *< 0.05, Fisher's exact test; Figure 1D and Table 1). Detoxification is one of the functions of the liver. Nuclear mt genes with liver-hypo T-DMRs also included the genes related to liver-specific functions, namely, *Otc *and *Lrpprc *(Table 1). OTC functions in the urea cycle in the liver [20]. LRPPRC is a binding partner of PPARGC1A, and functions in hepatic gluconeogenesis [21]. Nuclear mt genes with T-DMRs contained a number of genes involved in various types of mitochondrial regulation, such as the regulation of protein sorting, mitochondrial morphology, and translation of mtDNA-encoded proteins (Table 1).

**Table 1:** Nuclear mt genes with hypo T-DMRs

	Liver-hypo T-DMRs	Brain-hypo T-DMRs	Heart-hypo T-DMRs
Various mitochondrial functions			

Metabolism	*Abat, Acsl5, Oat, etc*	*Dmgdh, Mthfd1, Star, etc*	*Cpt1b, Efta, Pdk4, etc*

Respiratory chain	*Atp5o, Ndufb2, etc*	*Atp5l, Cox6, Ndufs8, etc*	*Atp5l, Ndufs4, Sdhc, etc*

TCA cycle	*Dlst*	*Aco2, Dlst*	*Ogdh*

Detoxification	*Gsr, Hagh, Mgst1, Tst*	*Gstk1*	

Tissue-specific mitochondrial functions	*Otc, Lrpprc*		

Protein sorting	*Immp1l*	*Tomm7*	

Regulation of mitochondrial morphology		*Mfn1, Prelid1*	

Regulation of mtDNA	*Peo1*		

Translation of mtDNA-encoded proteins	*Lrpprc*	*Mtif2, Mtif3*	*Mrrf, Tufm*

Human homologs of mouse nuclear mt genes possessing T-DMRs are related to various human diseases. For example, mutations of *Lrpprc*, *Ndufs4*, and *Ndufs8*, the genes with liver-, brain-, and heart-hypo T-DMRs, respectively, are associated with Leigh's disease [21-23]. In addition to mutations, overexpression of some nuclear mt genes with liver-hypo T-DMRs in non-liver tissues are involved in the human diseases. For example, overexpression of *Acsl5 *and *Tgm2 *are found in the human glioma and brain of Huntington's disease and are suggested to be involved in pathogenesis [24,25].

### Correlation between the DNA methylation status of T-DMRs and nuclear mt gene expression

**Figure 2:**
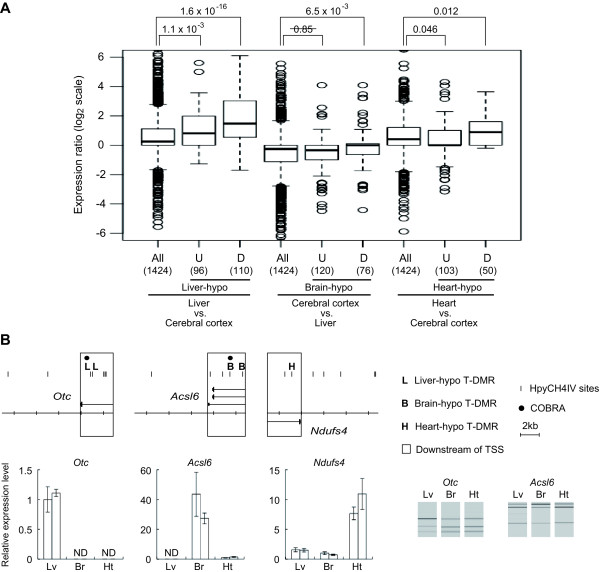
**Expression of nuclear mt genes with hypo T-DMRs**. (A) Boxplots show the expression ratio (log_2 _ratio) of the nuclear mt genes. Tissues used for comparisons are shown under the plots. "All" indicates that the plot shows the expression ratio of entire nuclear mt genes. "U" and "D" indicate that the plot shows the expression ratio of nuclear mt genes with hypo T-DMRs in upstream regions and downstream regions, respectively. The number of probe sets representing the expression levels of corresponding genes are displayed under the plot. *P*-values obtained from the Wilcoxon test are indicated on the top of the plot. (B) Genes which were relatively highly expressed in the tissues where downstream T-DMRs were hypomethylated, according to gene expression array data. Relative gene expression was confirmed using real-time PCR and was normalized to the expression of ß-actin (*Actb*). Bar graphs show the results of real-time PCR. "ND" indicates that the expression was not detected under the experimental condition used. Experiments were performed in biological duplicates, and all amplification were performed in triplicates. Error bars show standard deviations. For the *Otc *and *Acsl6 *genes, methylation levels of downstream hypo T-DMRs were examined by COBRA, and the electrophoresis images obtained by MultiNA microchip electrophoresis system are shown.

Next, we examined the correlation between the DNA methylation status of nuclear mt genes and their expression using microarray data downloaded from Gene Expression Omnibus (Figure 2A and Additional file 5). The liver-hypo T-DMRs positively correlated with the liver vs. cerebral cortex and liver vs. heart expression ratios (Figure 2A). In the brain and heart, only the hypo T-DMRs located downstream of TSSs positively correlated with the ratio of the expression in hypomethylated tissue vs. other tissue (Figure 2A and Additional file 5). On the other hand, negative correlation was observed between the upstream heart-hypo T-DMRs and the heart vs. cerebral cortex expression ratio, although the average expression levels were greater in the heart (Figure 2A). Thus, the downstream hypo T-DMRs correlated with the expression of the nuclear mt genes in all tissues examined. The correlation between T-DMRs around TSSs and tissue-specific gene expression coincided with that in the previous reports [11,15,26,27]. We confirmed the tissue-dependent gene expression of *Otc*, *Acsl6*, and *Ndufs4*, the genes carrying downstream hypo T-DMRs for liver, brain, and heart, respectively, by using real-time PCR (Figure 2B).

### Concentration of liver-hypo T-DMRs in the downstream regions of nuclear mt genes

**Figure 3:**
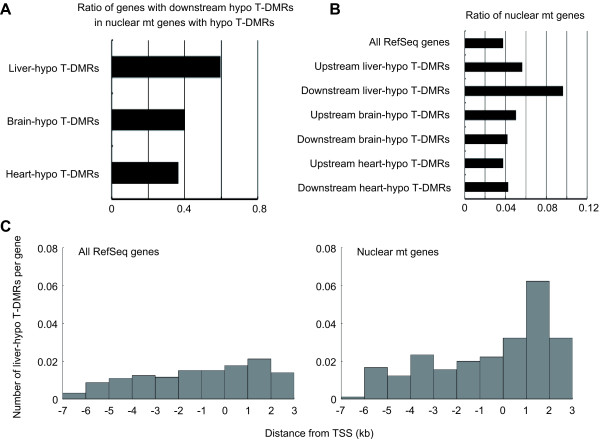
**Concentration of liver-hypo T-DMRs in downstream regions of nuclear mt genes**. (A) Bar graphs show the ratio of genes with downstream hypo T-DMRs in nuclear mt genes with hypo T-DMRs. (B) Bar graphs show the ratio of nuclear mt genes in all genes and genes with hypo T-DMRs. (C) The vertical axis of the histograms indicate the average number of liver-hypo T-DMRs per gene. Gene sets represented in histograms are indicated on the top-left of each graph.

In addition to the highest correlation of downstream liver-hypo T-DMRs with nuclear mt gene expression (Figure 2A and Additional file 5), a significantly larger proportion of the nuclear mt genes with liver-hypo T-DMRs contained these hypo T-DMRs in their downstream regions as compared to the nuclear mt genes with brain- and heart-hypo T-DMRs (*P *< 5 × 10^-3^, Fisher's exact test; Figure 3A). Furthermore, in genes with downstream liver-hypo T-DMRs, the nuclear mt genes were highly enriched (Figure 3B). Liver-hypo T-DMRs were especially enriched within regions of +1~+2 kb of TSSs of nuclear mt genes (Figure 3C). These results indicate that regulation of nuclear mt genes is especially dependent on DNA methylation in the liver.

### Overrepresented transcriptional regulatory motifs in nuclear mt genes with liver- and brain-hypo T-DMRs

We next investigated whether T-DMRs of nuclear mt genes are associated with any transcriptional regulatory motifs. FOXA2 is a transcription factor that activates the genes involved in mitochondrial β-oxidation and the regulation of lipid metabolism, ketogenesis, and insulin sensitivity in the mouse liver [28,29]. We analyzed the enrichment of genes containing FOXA2-binding sites within an extended gene region (encompassing 10 kb upstream of TSS and 1 kb downstream of the 3' UTR) using known genome-wide FOXA2-binding sites in the mouse liver obtained by ChIP-sequencing [30]. We found that the targets of FOXA2 were enriched 1.6-fold in nuclear mt genes with liver-hypo T-DMRs relative to all nuclear mt genes (*P *< 5 × 10^-5^, Fisher's exact test). Sixty-four out of 123 nuclear mt genes with liver-hypo T-DMRs were the targets of FOXA2 (Additional file 6). This is contrast to the previous report, which indicated enrichment of target genes of HNF1 and/or HNF4 in hypomethylated genes in the mouse liver [11].

We further analyzed the enrichment of specific regulatory motifs in nuclear mt genes with hypo T-DMRs by using the oPOSSUM program [31]. We used all nuclear mt genes as a background gene set, and analyzed both 5 kb upstream and downstream of TSSs of the genes with hypo T-DMRs. The enrichment of the genes with FOXA2-binding sites in their downstream region was observed in the nuclear mt genes with liver-hypo T-DMRs. (Table 2 and Additional file 7). It has been reported that FOXA2-binding at distal downstream region of TSS of *CEBPA *gene functions as an enhancer in humans [32]. Hypomethylation of downstream T-DMRs may enhance transcription by permitting transcription factors access to target genes. We also found that genes with NFYA-binding sites in their downstream regions were enriched among the nuclear mt genes with liver-hypo T-DMRs, and the genes with CEBPA- and STAT1-binding sites in their upstream regions were enriched among the nuclear mt genes with brain-hypo T-DMRs (Table 2 and Additional file 7). These results indicate that T-DMRs are associated with specific transcription factors in a tissue-dependent manner. CEBPA and STAT1 are reported to be involved in cortical neurogenesis [33] and inflammatory responses in the brain [34], respectively. It has been reported that CEBPA-null mice showed decreased nuclear mt gene expression in brown adipose tissue [35]. STAT1 also regulates nuclear mt gene expression in T cells in response to IFN-γ [36]. Hypomethylation of hypo T-DMRs of targets of these transcription factors may permit transcription factors access to the target genes, and hypermethylation of these T-DMRs may be essential for secured repression of the corresponding genes in other tissues.

**Table 2:** Transcription factors whose binding-sites were enriched in analyzed sequences

Gene set	Analyzed sequences	Transcription factor	Number of targets	Enrichment	*P *value*
Liver-hypo	5 kb-downstream	FOXA2	52	1.23	3.1 × 10^-2^
		NFYA	29	1.44	3.6 × 10^-2^
Brain-hypo	5 kb-upstream	CEBPA	59	1.21	2.3 × 10^-2^
		STAT1	25	1.52	2.5 × 10^-2^

* *P *values represent results of Fisher's exact test.

## Conclusion

At least 71% of investigated nuclear mt genes contains T-DMRs, and the methylation status of T-DMRs correlated with tissue-dependent expression of dozens of nuclear mt genes. Considering that there are at least 200 different cell types in the mammalian body, the total number of nuclear mt genes with T-DMRs will be higher. The differences in protein composition of mitochondria are reported to reflect tissue-dependent nuclear mt gene expression [37]. Our data suggest that DNA methylation status of nuclear mt genes underlies tissue-dependent mitochondrial functions.

## Methods

### Mice

C57BL/6N male mice were obtained from Charles River Japan. Mice were euthanized at 12-13 week old, and tissues were collected and frozen at -80°C until use. All experiments using mice were carried out according to the institutional guidelines for the care and use of laboratory animals (Graduate School of Agriculture and Life Sciences, The University of Tokyo).

### Genomic DNA extraction

Genomic DNA was extracted as described previously [11]. Briefly, tissue samples were homogenized, and incubated with lysis solution (10 mM Tris-HCl at pH 8.0, 5 mM EDTA, 200 mM NaCl, 0.2% SDS, and 200 μg/mL proteinase K) at 55°C for 30 min, and were extracted with a phenol/chloroform/isoamylalcohol (PCI) mixture (50 : 49 : 1), incubated with RNase for 30 min, and re-extracted with PCI. DNA was precipitated with ethanol and dissolved in Tris-EDTA (TE) buffer (pH 8.0).

### D-REAM analysis

We used T-DMR profiling with restriction-tag mediated amplification (D-REAM) analysis [11] to obtain tissue-dependent and differentially methylated HpyCH4IV sites within the TSS flanking regions of RefSeq genes. For D-REAM analysis, HpyCH4IV-digested genomic DNA was extracted with PCI, re-extracted with chloroform, precipitated with ethanol and dissolved in TE (pH 8.0). Using purified DNA (250 ng), following procedure of D-REAM analysis was performed as described previously [11]. Briefly, genomic DNA was digested by the methyl-sensitive enzyme HpyCH4IV (New England Biolabs), followed by ligation-mediated PCR, and subsequent hybridization of DNA to a GeneChip Mouse Promoter 1.0R Array (Affymetrix). Comparison of the resulting signals from digested HpyCH4IV sites between different tissue samples indicates the differential methylation level at a given site.

D-REAM analysis was performed twice for each of the biological duplicates of heart, and once for the liver and brain in this study. For the liver and brain, we added single D-REAM data set from our previous study using tissues from different individual [11]. Correlation coefficients of microarray probe intensities between biological duplicates were greater than 0.93. D-REAM data obtained in this study has been deposited in the ArrayExpress database (accession number A-MEXP-791).

### Combined bisulfite restriction analysis (COBRA)

Genomic DNA was digested with HindIII (Takara). Digested DNA (5 μg) was denatured with 0.3 M NaOH. Sodium metabisulfite (pH 5.0) and hydroquinone were added to a final concentration of 2.0 M and 0.5 mM, respectively. The reaction mixture was incubated under following conditions: 15 cycles of 95°C for 30 s and 50°C for 15 min. Next, 1.77 volume of QG buffer was added to the reaction mixture, and DNA was purified using a Quiagen gel extraction kit (Qiagen), and eluted with 100 μl of elution buffer (EB). DNA was treated with 0.3 M NaOH at 37°C for 15 min, precipitated using 6 M ammonium acetate (pH 7.0) and ethanol, and dissolved in 200 μl TE (pH 8.0). For each bisulfite PCR, 2 μl of DNA solution was used as the template, and BIOTAQ HS DNA polymerase (Bioline) was used for amplification. PCR was performed under the following conditions: denaturation at 95°C for 10 min followed by 43 cycles, each cycle comprising 95°C for 30 sec, 60°C for 45 sec, 72°C for 30 sec, followed by 10 min at 72°C. All primers used in this experiment are listed in Additional file 2. The PCR product was digested with HpyCH4IV. Restriction-enzyme-treated DNA was desalted using gel filtration through Sephadex G-50, and was analyzed using the MultiNA microchip electrophoresis system (Shimadzu). The methylation level was calculated as the ratio of the amounts of cut fragments to those of the total of cut and uncut fragments obtained from the electropherograms.

### RNA extraction, reverse transcription, and real-time PCR

Total RNA was prepared using TRIzol reagent (Invitrogen). Before synthesis of first-strand cDNA, the RNA preparation was treated with RNase-free DNase I (Invitrogen) to eliminate any residual genomic DNA. The total RNA was then converted into first-strand cDNA using random hexamers and Superscript III First-Strand Synthesis System for RT-PCR (Invitrogen). The obtained cDNA were amplified and quantified in triplicates by using the Quantitect SYBR Green PCR Kit (Qiagen) with ABI 7500 Real Time PCR system (Applied Biosystems). PCR was performed under the following conditions: Incubation at 95°C for 10 min followed by 40 cycles of PCR, each cycle comprising 95°C for 15 sec and 60°C for 1 min. All primers used in this experiment are listed in Additional file 8. Standard curves were obtained with serial dilutions of a pool of cDNA samples derived from each tissue.

### Bioinformatics

MAT [18] was used to analyze the tiling array data (.CEL files) and identify the hypomethylated regions based on tiling probe signals, probe sequences, and copy numbers. Original tiling probes were remapped to the mouse genome assembly version mm9 (July 2007 build) provided by UCSC genome database. For the quality control of D-REAM analysis, we monitored the selective amplification of HpyCH4IV-digested fragments for the tilling array data of each sample (Additional file 9).

For expression analysis, data from the GeneChip Mouse Genome 430 2.0 Array of liver, heart, and cerebral cortex tissues of C57BL/6N male mice (8-10 week old; *n *= 2 for each tissue) were downloaded from Gene Expression Omnibus (accession no. GSE10246). The array image data (.CEL files) was processed by the factor analysis for robust microarray summarization algorithm (FARMS) with quantile normalization [38].

Enrichment analysis of specific transcription factor targets was performed using oPOSSUM program [31]. This program analyzed the genes using one-to-one human-mouse orthologs and detected promoter motifs in the conserved regions. The top 10% of the non-coding conserved regions with an absolute minimum percent identity of 70% in each 5 kb region upstream and downstream of the TSSs were analyzed for vertebrate promoter motifs with a matrix match threshold of 75%.

## Authors' contributions

MT and KS designed this study. KH performed D-REAM. MT performed COBRA and data analysis with help of SY, and wrote the paper with SY and KS. All authors read and approved the final manuscript.

## Supplementary Material

Additional file 1 **Figure S1 Differential methylation levels of HpyCH4IV sites and the corresponding MATscores**. Click here for file

Additional file 2 **Table S1 Primers used for COBRA, and the result of the experiment**. Click here for file

Additional file 3 **Table S2 Genomic locations of T-DMRs**. Click here for file

Additional file 4 **Table S3 Genomic locations of hypo T-DMRs**. Click here for file

Additional file 5 **FIgure S2 Expression ratio of nuclear mt genes with hypo T-DMRs**. Click here for file

Additional file 6 **Table S4 Genes with liver-hypo T-DMRs and FOXA2-binding sites**. Click here for file

Additional file 7 **Table S5 Genes with hypo T-DMRs and overrepresented transcription factor binding sites**. Click here for file

Additional file 8 **Table S6 Primers used for real-time PCR**. Click here for file

Additional file 9 **Figure S3 Distribution of MATscores calculated from D-REAM data of each tissue**. Click here for file
