# Supplementary material for: DNA methylation status of nuclear-encoded mitochondrial genes underlies the tissue-dependent mitochondrial functions
Source: BMC Genomics. 2010 Aug 19;11:481. doi: 10.1186/1471-2164-11-481 (PMC2996977; doi:10.1186/1471-2164-11-481)
Supplement: Additional file 1 — Figure S1 Differential methylation levels of HpyCH4IV sites and the corresponding MATscores. [file 1471-2164-11-481-S1.PDF]

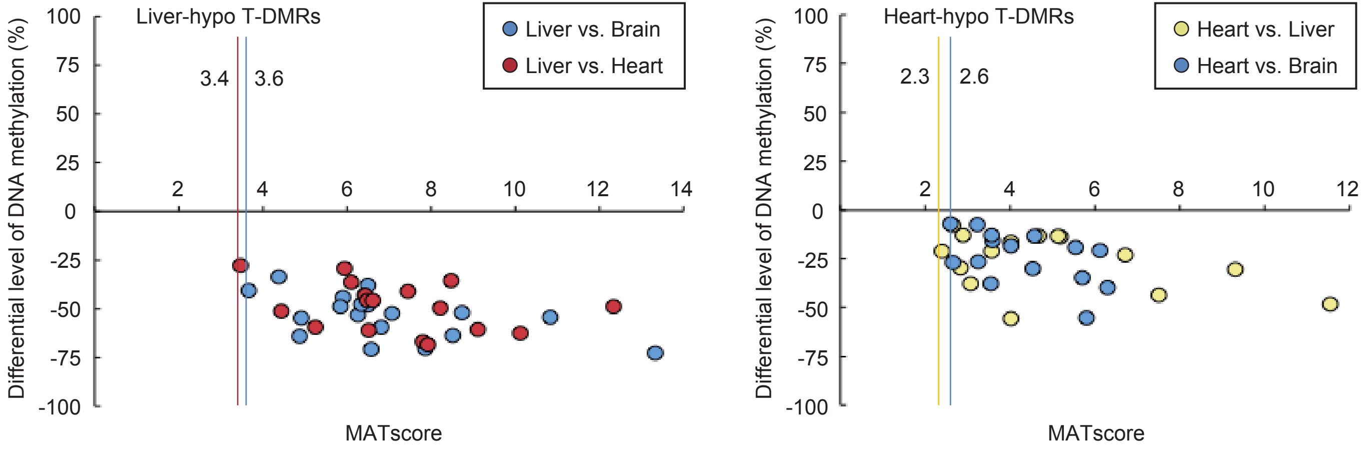

Figure S1 Differential methylation levels of HpyCH4IV sites and the corresponding MATscores. Differential methylation levels of HpyCH4IV sites for which the relative hypomethylation levels were analyzed using the D-REAM analysis. For each T-DMR, the corresponding MATscore is indicated along the horizontal axis. In each tissue comparison, the lowest MATscore of the HpyCH4IVsites, whose hypomethylation were confimed using COBRA, was considered as a threshold value for detecting hypomethylated regions in the tissue comparison.
