# Supplementary material for: DNA methylation status of nuclear-encoded mitochondrial genes underlies the tissue-dependent mitochondrial functions
Source: BMC Genomics. 2010 Aug 19;11:481. doi: 10.1186/1471-2164-11-481 (PMC2996977; doi:10.1186/1471-2164-11-481)
Supplement: Additional file 5 — FIgure S2 Expression ratio of nuclear mt genes with hypo T-DMRs. [file 1471-2164-11-481-S5.PDF]

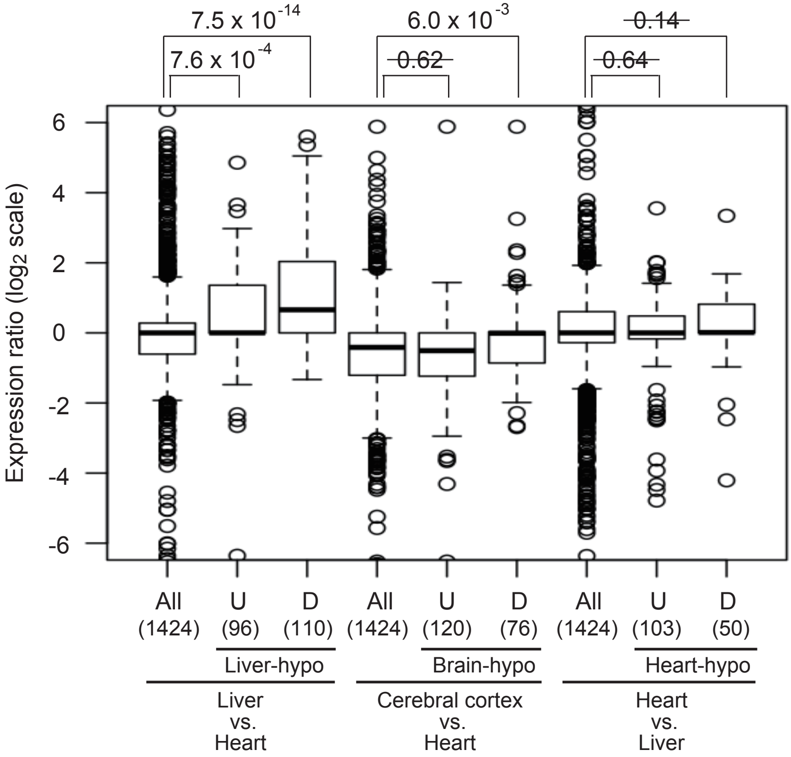

Figure S2 Expression ratio of nuclear mt genes with hypo T-DMRs. Boxplots show the expression ratio (log_2 ratio) of the nuclear mt genes. Tissues used for comparisons are shown under the plots. Only comparisons not shown in Figure 2 are shown in this figure. "All" indicates that the plot shows the expression ratio of whole nuclear mt genes. "U" and "D" indicate that the plot shows the expression ratio of nuclear mt genes with hypo T-DMRs in upstream regions and downstream regons, respectively. Number of probe sets representing the expression levels of corresponging genes are displayed under the plot. *P*-values obtained from the Wilcoxon test are indicated on the top of the plot.
