# Supplementary material for: DNA methylation status of nuclear-encoded mitochondrial genes underlies the tissue-dependent mitochondrial functions
Source: BMC Genomics. 2010 Aug 19;11:481. doi: 10.1186/1471-2164-11-481 (PMC2996977; doi:10.1186/1471-2164-11-481)
Supplement: Additional file 9 — Figure S3 Distribution of MATscores calculated from D-REAM data of each tissue. [file 1471-2164-11-481-S9.PDF]

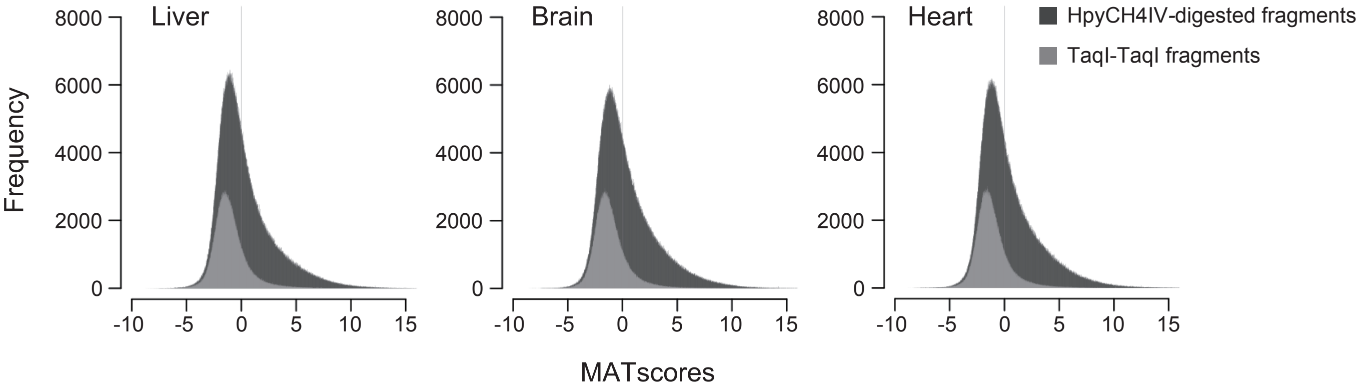

Figure S3 Distribution of MATscores calculated from D-REAM data of each tissue. MATscore distribution of array regions corresponding to the TaqI-TaqI fragments (gray) and HpyCH4IV-digested fragments (black) are indicated.
